# Supplementary material for: Identification of photoperiod-induced specific miRNAs in the adrenal glands of Sunite sheep (Ovis aries)
Source: Front Vet Sci. 2022 Jul 22;9:888207. doi: 10.3389/fvets.2022.888207 (PMC9354845; doi:10.3389/fvets.2022.888207)
Supplement: Supplementary file 1 [file Data_Sheet_1.ZIP › Supplementary file/miRDeep2 file.zip!/miRDeep2 file/Know_Structure/SPLP42A1/oar-let-7g.pdf]

miRBase precursor : oar-let-7g
Total read count : 652090
oar-let-7g read count : 652081
remaining reads : 9

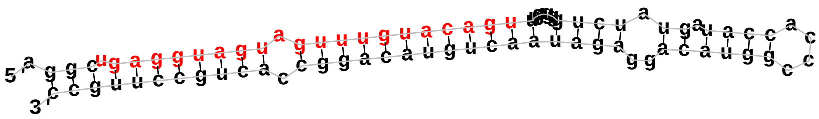

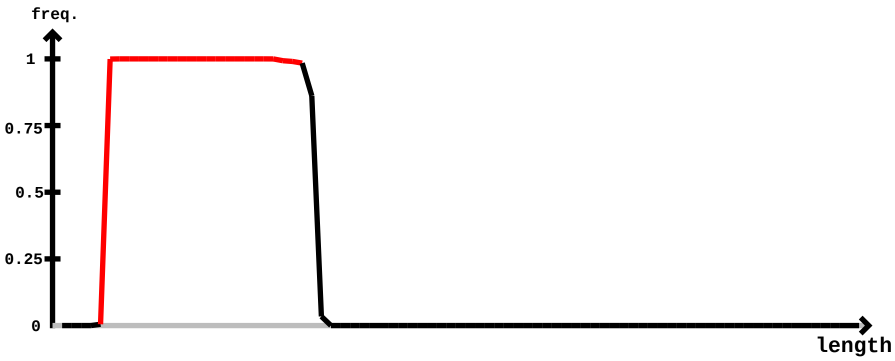

oar-let-7g

```
5'- aggcugagguaguaguuuguacaguuugagggucuaugauaccacccgguacaggagauaacuguacaggccacugccuugcc   -3'    exp
    .(((.((((((((.(((((((((((.....((((.((.((((....))))))..))))))))))))))).)))))))))))    reads   mm   sample
    ..gcugagguaguaguuuguacaguu.........................................................   1       0    seq
    ...cugagguaguaguuuguac.............................................................   32      0    seq
    ...cugagguaguaguuuguaca............................................................   14      0    seq
    ...cugagguaguaguuuguacag...........................................................   34      0    seq
    ...cugagguaguaguuuguacagu..........................................................   597     0    seq
    ...cugagguaguaguuuguacaguu.........................................................   2445    0    seq
    ...cugagguaguaguuuguacaguuu........................................................   91      0    seq
    ....ugagguaguaguuugua..............................................................   125     0    seq
    ....ugaAguaguaguuugua..............................................................   1       1    seq
    ....ugUgguaguaguuugua..............................................................   1       1    seq
    ....ugagguaguaguuuguac.............................................................   4317    0    seq
    ....ugagguagCaguuuguac.............................................................   9       1    seq
    ....ugagguaguaguuuguaca............................................................   1818    0    seq
    ....ugagguaguaguuuguacag...........................................................   4003    0    seq
    ....ugagguaguaguuuguacagu..........................................................   79128   0    seq
    ....ugagguaguaguuuguacaguu.........................................................   536778  0    seq
    ....ugagguaguaguuuguacaguuu........................................................   22274   0    seq
    ....ugagguaguaguuuguacaguuug.......................................................   11      0    seq
    .....gagguaguaguuuguac.............................................................   1       0    seq
    .....gagguaguaguuuguacag...........................................................   2       0    seq
    .....gagguaguaguuuguacagu..........................................................   44      0    seq
    .....gagguaguaguuuguacaguu.........................................................   335     0    seq
    .....gagguaguaguuuguacaguuu........................................................   17      0    seq
    ......agguaguaguuuguacaguu.........................................................   3       0    seq
    ............................................................acuguacaggccacugccuugc.   2       0    seq
    .............................................................cuguacaggccacugccuugc.   4       0    seq
    .............................................................cuguacaggccacugccuugcc   3       0    seq
```
